# ^1^H, ^13^C and ^15^N backbone resonance assignment of Cel45A from *Phanerochaete chrysosporium*

**DOI:** 10.1007/s12104-024-10182-6

**Published:** 2024-06-18

**Authors:** Laura Okmane, Mats Sandgren, Jerry Ståhlberg, Gustav Nestor

**Affiliations:** Department of Molecular Sciences, Swedish University of Agricultural Sciences, Uppsala, Sweden

**Keywords:** *Pc*Cel45A, Cel45A, GH45, Endoglucanase, NMR

## Abstract

A glycoside hydrolase family 45 (GH45) enzyme from the white-rot basidiomycete fungus *Phanerochaete chrysosporium* (*Pc*Cel45A) was expressed in *Pichia pastoris* with ^13^C and ^15^N labelling. A nearly complete assignment of ^1^H, ^13^C and ^15^N backbone resonances was obtained, as well as the secondary structure prediction based on the assigned chemical shifts using the TALOS-N software. The predicted secondary structure was almost identical to previously published crystal structures of the same enzyme, except for differences in the termini of the sequence. This is the first NMR study using an isotopically labelled GH45 enzyme.

## Biological context

Glycoside hydrolase family 45 (GH45) enzymes are inverting endoglucanases which exhibit cellulase (EC 3.2.1.4), endo-xyloglucanase (EC 3.2.1.151) or endo-β-1,4-mannanase (EC 3.2.1.78) activity. The catalytic domain (CD) has a double-psi-beta barrel (DPBB) fold. Sequence-based phylogenetic analyses divide GH45s into three subfamilies: A, B, and C. The majority of thus far studied GH45 enzymes belong to subfamily A. The least studied but perhaps the most perplexing due to its atypical catalytic site is subfamily C, with Cel45A from the white-rot basidiomycete fungus *Phanerochaete chrysosporium* (*Pc*Cel45A) as the first characterized member of subfamily C. Subfamily C members exhibit relatively low catalytic activities and possess a structural resemblance to non-hydrolytic protein groups named expansins, expansin-like proteins, loosenins, and swollenins (Cosgrove 2015).

The major divisible differences within the GH45 family are in respect to the catalytic residues. The catalytic acid is conserved in all subfamilies but the catalytic base seems to be absent in subfamily C. Subfamily A and B enzymes utilize two aspartic acid residues to act as a catalytic acid and a catalytic base. GH45 subfamily C members have conserved the aspartic acid residue as a catalytic acid but the catalytic base remains undiscovered (Igarashi et al. 2008), therefore the factual hydrolytic mechanism of GH45 subfamily C members is unknown. Interestingly, the lack of an acidic residue at the supposed catalytic base position does not lead to inactivity of the subfamily C members. This has encouraged the proposition of a novel action mechanism in *Pc*Cel45A (Nakamura et al. 2015), where the imidic acid form of Asn-92 acts as the catalytic base. Regarding the catalytic mechanism, the subfamily C thus appears distinctively different from GH45 subfamilies A and B, still subfamily C and B are more alike in regard to reaction product profile (Okmane et al. 2022). To this date, two GH45 subfamily C enzymes have been crystallized – *Pc*Cel45A, PDB ID 3X2O (Nakamura et al. 2015) and 5KJO (Godoy et al. 2018), and Cel45A from *Gloeophyllum trabeum*, PDB ID 8BZQ (Okmane et al. 2024). Crystal structures of *Pc*Cel45A in complex with cellobiose, PDB ID 5KJQ (Godoy et al. 2018), and with cellopentaose, PDB IDs 3X2P, 3X2M (Nakamura et al. 2015), are publicly available. No structural studies by NMR spectroscopy on *Pc*Cel45A or any other GH45 enzyme have been published previously. All structural information thus far is derived from x-ray crystallography and neutron crystallography.

Classically, labelled protein expression for NMR studies is carried out using *E. coli* platform strains. In this study, we (1) describe the production of a ^13^C and ^15^N-labelled *Pc*Cel45A expressed in *Pichia pastoris*; (2) report an almost complete assignment of the *Pc*Cel45A ^1^H, ^13^C and ^15^N backbone resonances; (3) compare secondary structural elements derived from the backbone resonances of *Pc*Cel45A to a crystal structure of the same enzyme.

## Methods and experiments

### Protein expression and purification

Heterologous expression of *Pc*Cel45A (GenBank: BAG68300) was done in *P. pastoris* KM71H expression system according to Igarashi et al. (2008).

For the production of the isotopically labelled *Pc*Cel45A, a two-phase fermentation scheme was employed in Infors HT Multifors 2 bioreactors. In the first phase (biomass accumulation), an inoculum (5 mL of OD = 1, 30 °C; 180 rpm, same medium as during cultivation) was used to initiate the batch-culture of *P. pastoris* strain KM71H in minimal medium. The minimal medium was based on a protocol from Courtade et al. (2016) with some modifications and was composed of 0.34% yeast nitrogen base (YNB) without amino acids or (NH_4_)_2_SO_4_, 1% (^15^NH_4_)_2_SO_4_, 4 × 10^− 5^% biotin, 1.5% ^13^C_6_-glucose, and 400 µL Antifoam B. Aeration was set to 500 rpm, pO_2_ min 22%, and constant temperature at 25 °C and a pH of 5. Regulation of pH was done with 3 M H_3_PO_4_ and 5 M NaOH. Glucose levels were monitored by sampling and glucose concentration was determined using Medi-Test Glucose strips (Macherey-Nagel). Upon full glucose depletion, the second phase (induction phase) was started by lowering the temperature to 20 °C and adding methanol (25% ^13^C-labelled) at a feed rate of 0.38 mL/h. Induction was terminated and cultures were harvested after 48 h. Cultures were centrifuged for 10 min at 4000 rpm, the supernatant was filtered (0.45 μm and 0.2 μm PES sterile filters) and concentrated using Vivaspin 5MWCO centrifugal concentration devices (Sartorius).

The protein purification was initiated by a buffer exchange into 20 mM Tris pH 7 buffer using a Biogel P6 desalting column, followed by anion exchange using a DEAE Sepharose FF column and the following buffers: (A) 20 mM Tris, pH 7.0; (B) 20 mM Tris, 0.5 M NaCl, pH 7.0. Approximately 6 mg of protein was loaded onto a gel filtration column Superdex 75 16/600, 50 mM K-phosphate buffer pH 5.5, 0.1 M NaCl. Purification from 400 mL culture filtrate yielded 2.5 mg pure protein. Protein concentrations were determined spectrophotometrically at 280 nm with an extinction coefficient of 23,085 M^-1^ cm^-1^. Eventually, a pure protein sample was concentrated to 450 µM in 50 mM K-phosphate buffer, pH 5.5.

### NMR spectroscopy

NMR experiments were performed on a Bruker Avance III 600 MHz spectrometer with a 5 mm ^1^H/^13^C/^15^N/^31^P inverse detection cryoprobe equipped with z gradient. Spectra were recorded at 25 °C and were processed with TopSpin 4.1.4. Assignments of sequence-specific ^1^H, ^13^C and ^15^N backbone resonances were obtained from 2D ^1^H,^15^N-HSQC and 3D HNCA, HNCACB, CBCA(CO)NH, HNCO, HN(CA)CO, and HBHA(CO)NH spectra. ^1^H and ^13^C chemical shifts were referenced directly to internal DSS-*d*_6_ and ^15^N chemical shifts were referenced indirectly using frequency ratios (Wishart et al. 1995). Spectra were analyzed manually using CcpNmr Analysis 3.1.1 (Skinner et al. 2016). Secondary structural elements were predicted based on the NMR data using TALOS-N (Shen and Bax 2013) with the backbone N, HN, Cα, Cβ, Hα, and C′ chemical shifts as input. The secondary structure predicted by TALOS-N was compared with a combined neutron/X-ray diffraction crystal structure of *Pc*Cel45A with the PDB accession code 3X2O (Nakamura et al. 2015).

## Extent of assignment and data deposition

### ^13^C and ^15^N isotopic labelling

The sole nitrogen source during cell cultivation and protein expression was 1% (^15^NH_4_)_2_SO_4_. The carbon source was exchanged from 1.5% ^13^C_6_-glucose during cell cultivation to methanol (25% ^13^C-labelled) during protein expression. This approach yielded *Pc*Cel45A with > 95% ^15^N labelling and ~ 70% ^13^C labelling. The 70% ^13^C labelling did not have a significant effect on the sensitivity of multi-step NMR experiments, such as HNCACB. The main carbon source was most likely glucose (99% ^13^C-labelled), whereas the methanol (25% ^13^C-labelled) is responsible for the decrease to 70% labelling. Since glucose is the main carbon source, the distribution of ^13^C is not random and the probability that two linked carbon atoms are labelled is thus larger than 0.7 × 0.7 = 0.49. This could explain the negligible impact on NMR experiments involving carbon-carbon transfers.

### Backbone resonance assignment of PcCel45A

**Fig. 1:**
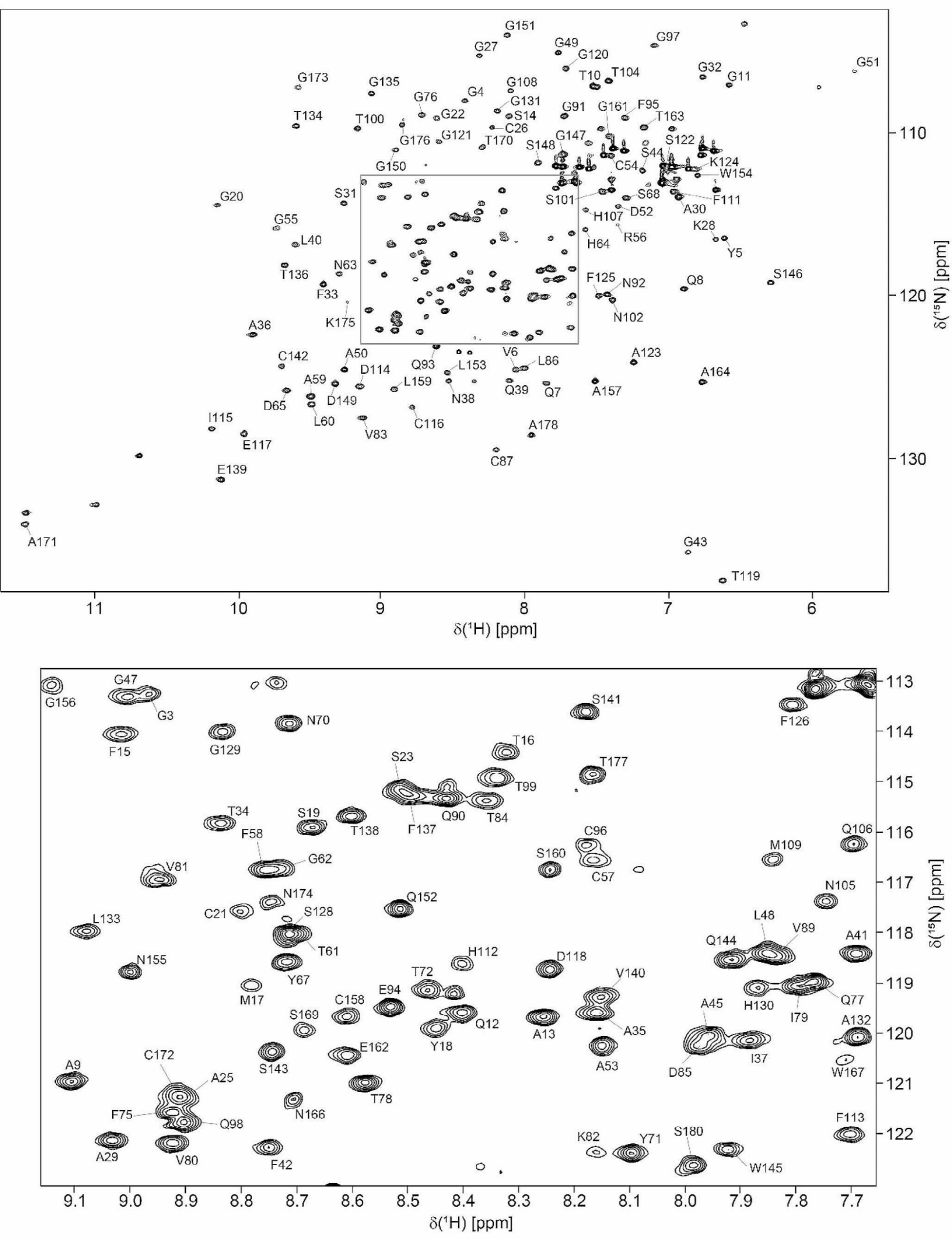
^1^H,^15^N-HSQC spectrum of *Pc*Cel45A with an expansion of the central region shown below and marked as a grey box in the upper spectrum. The spectrum was acquired from ^15^N-labelled *Pc*Cel45A (90 µM) in 50 mM potassium phosphate buffer, pH 5.0 and 90% H_2_O/10% D_2_O on a 600 MHz spectrometer at 25 °C. The two residues with the seemingly highest ^15^N chemical shift (G43 and T119) were folded in the spectrum and have ^15^N chemical shifts of 97.74 and 99.47 ppm, respectively

The 2D ^1^H,^15^N-HSQC spectrum of *Pc*Cel45A exhibited well dispersed and narrow cross-peaks indicative of a native folded structure (Fig. 1). *Pc*Cel45A is an 18 kDa protein composed of 180 residues, including 12 prolines and 30 glycines. Due to the high number of glycines with similar Cα chemical shifts, we used a combination of 3D HNCACB and CBCA(CO)NH for sequential assignment of Cα and Cβ, and 3D HNCO and HN(CA)CO for sequential assignment of C′. All expected amide backbone resonances were identified except for Thr2 and Gly73. In addition, Hα resonances were assigned with the help of a 3D HBHA(CO)NH spectrum.

### Secondary structure of PcCel45A in solution

**Fig. 2:**
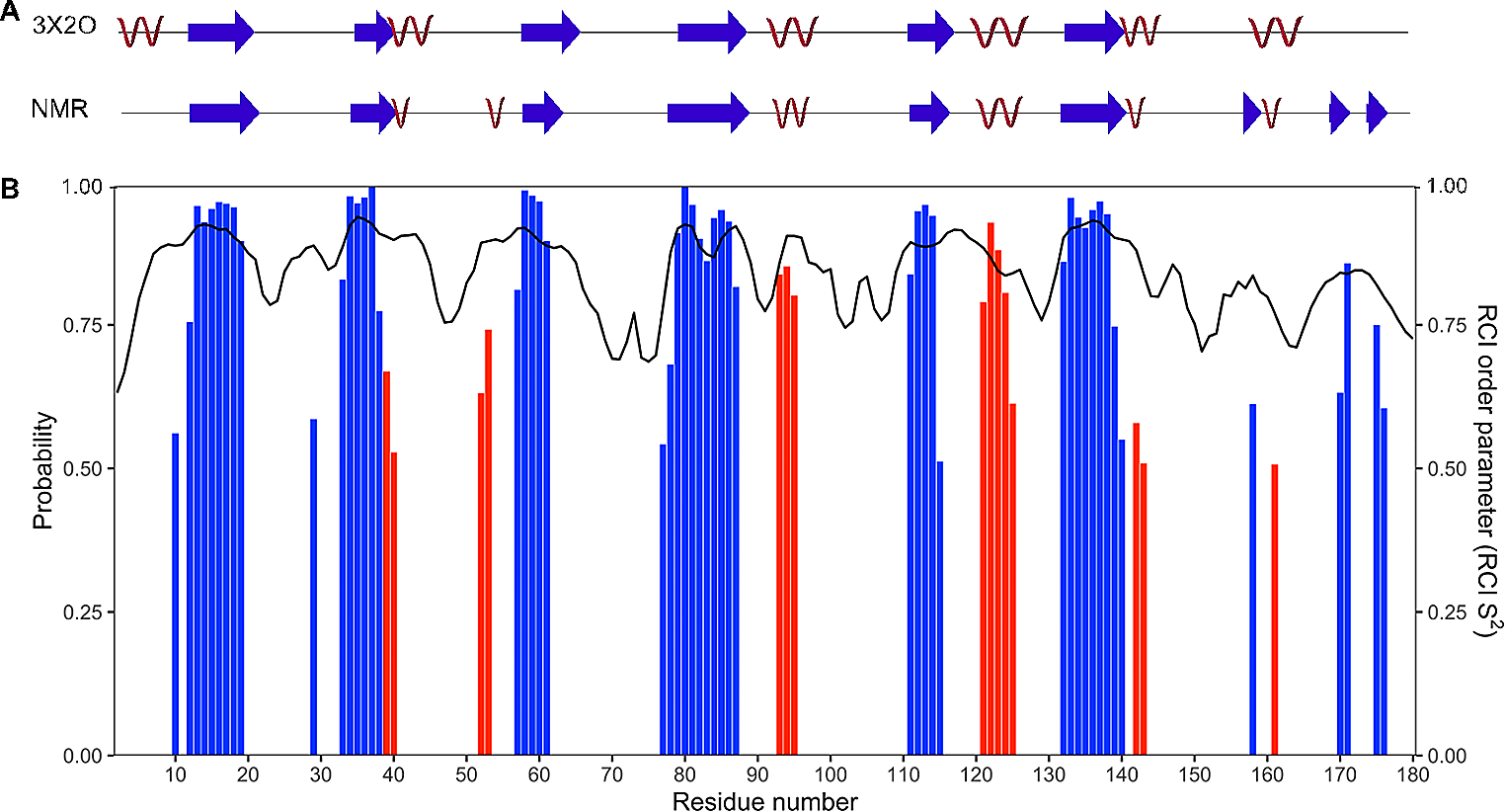
Secondary structure and RCI order parameters (S^2^) of *Pc*Cel45A calculated on the basis of the assigned backbone chemical shifts. **(A)** Schematic diagram of *Pc*Cel45A secondary structure (red helices; blue arrows, β-sheets) with a comparison of the NMR-derived secondary structure with the crystal structure 3X2O. **(B)** The predicted secondary structure (red, helix; blue, β-sheet) is shown with the height of the bars corresponding to the probability assigned by TALOS-N. RCI S^2^ order parameters for each residue calculated by TALOS-N are shown as a black line

The secondary structure of *Pc*Cel45A in solution was predicted using the TALOS-N software based on the chemical shift data from the current study (Fig. 2). The random coil index (RCI) order parameter S^2^ (Berjanskii and Wishart 2008) shows relatively high numbers (> 0.6) for all the residues, indicating a well folded structure. Dips in RCI S^2^ values are consistent with less ordered loops, which are found in-between sheets and helices. The secondary structure elements were compared with a previously published crystal structure of *Pc*Cel45A (Nakamura et al. 2015), showing clear similarities, but with some distinct differences: Starting from the N-terminal, residue 1–5 form a helix in the crystal structure, which is not observed in the NMR derived secondary structure. The NMR structure shows helical propensity for residue 52–53, which is absent in the crystal structure. Similarly, the C-terminal part of *Pc*Cel45A (residue 170–176) form a pair of β-strands based on the NMR data, but is considered to be relatively disordered in the crystal structure. These differences are all limited to residues far from the catalytic site of *Pc*Cel45A and are likely not affecting the substrate binding or the catalytic activity to a large extent.

## Data Availability

The assignments for the backbone resonances have been deposited at the Biological Magnetic Resonance Data Bank under the accession number 52371.
